# The Parasol Protocol: An Implementation Science Study of HIV Continuum of Care Interventions for Gay Men and Transgender Women in Burma/Myanmar

**DOI:** 10.2196/resprot.7642

**Published:** 2017-05-17

**Authors:** Andrea L Wirtz, Soe Naing, Emily Clouse, Kaung Htet Thu, Sandra Hsu Hnin Mon, Zin Min Tun, Stefan Baral, Aung Zayar Paing, Chris Beyrer

**Affiliations:** ^1^ Center for Public Health and Human Rights Department of Epidemiology Bloomberg School of Public Health, Johns Hopkins University Baltimore, MD United States; ^2^ International HIV/AIDS Alliance in Myanmar Yangon Myanmar

**Keywords:** Myanmar, men who have sex with men, transgender, HIV, antiretroviral therapy

## Abstract

**Background:**

Efforts to improve HIV diagnosis and antiretroviral therapy (ART) initiation among people living with HIV and reduce onward transmission of HIV rely on innovative interventions along multiple steps of the HIV care continuum. These innovative methods are particularly important for key populations, including men who have sex with men (MSM) and transgender women (TW). The HIV epidemic in Myanmar is concentrated among key populations, and national efforts now focus on reducing stigma and improving engagement of MSM and TW in HIV prevention and care.

**Objective:**

This study aims to test the use of several innovations to address losses in the HIV care continuum: (1) use of respondent-driven sampling (RDS) to reach and engage MSM and TW in HIV testing, (2) HIV self-testing (HIVST) to increase HIV testing uptake and aid early diagnosis of infection, (3) community-based CD4 point-of-care (POC) technology to rapidly stage HIV disease for those who are HIV infected, and (4) peer navigation support to increase successful health system navigation for HIV-infected MSM and TW in need of ART or HIV engagement in care.

**Methods:**

To assess the effect of HIVST, we will implement a randomized trial in which MSM and TW adults in the greater Yangon metropolitan area who are HIV uninfected will be recruited via RDS (N=366). Participants will complete a baseline socio-behavioral survey and will be randomized to standard, voluntary counseling and testing (VCT) or to HIVST. Biologic specimens will be collected during this baseline visit for confirmatory testing using dried blood spots. Participants will be asked to return to the study office to complete a second study visit in which they will report their HIV test result and answer questions on the acceptability of the assigned testing method. Aim 1 participants with confirmed HIV infection and who are not engaged in care (N=49) will be offered direct enrollment into Aims 2 and 3, which include immediate CD4 POC and the option for peer navigation, respectively. Aims 2 and 3 participants will be prospectively followed for 12 months with data collection including interviewer-administered sociobehavioral survey, CD4 POC, and viral load testing occurring biannually. Participants who accept peer navigation will be compared to those who decline peer navigation. Analyses will estimate the impact of CD4 POC on engagement in care and the impact of peer navigation on ART adherence and viral load.

**Results:**

Formative qualitative research was conducted in June and September 2015 and led to further refinement of recruitment methods, HIVST instructions and counseling, and peer navigation methods. Aim 1 recruitment began in November 2015 with subsequent enrollment into Aims 2 and 3 and is currently ongoing.

**Conclusions:**

These innovative interventions may resolve gaps in the HIV care continuum among MSM and TW and future implementation may aid in curbing the HIV epidemic among MSM and TW in Myanmar.

## Introduction

Efforts at both international and national levels have been re-energized toward ending the HIV epidemics. The United Nations and national strategies now focus on increased testing for the identification of previously undiagnosed HIV infection for linkage to care and treatment engagement [1]. Since the HIV Prevention Trials Network (HPTN) study 052 highlighted the preventive benefits of early antiretroviral therapy (ART) treatment [2], most countries have adapted their treatment criteria to initiate treatment at CD4 <500 cells/µL, in accordance with 2013 World Health Organization (WHO) guidelines, while some countries have begun adopting the 2015 WHO guidelines of providing treatment for all people living with HIV [3]. The majority of these global efforts focus on the general populations, although for settings where HIV is concentrated among key populations, such as gay men and other men who have sex with men (MSM), transgender women (TW), sex workers, and people who use drugs, it is imperative to ensure that access to treatment and care is available for key populations in order to have true impact on the epidemic trajectories [4,5].

**Figure 1:**
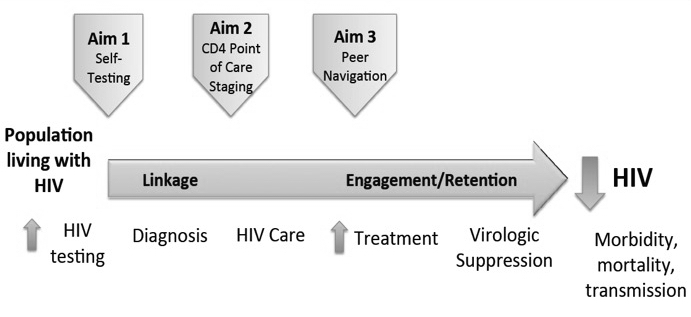
HIV care continuum among people living with HIV.

Achieving such goals for MSM and TW is challenging given contexts of stigma, discrimination, and criminalization and require innovative approaches to overcoming these challenges and engaging these populations in HIV testing and care. Considering the HIV care continuum (Figure 1), these innovative approaches can be viewed in terms of several stages for MSM and TW: (1) initial contact with or outreach to MSM and TW, (2) HIV testing and diagnosis, (3) HIV staging according to national criteria, (4) engagement and retention in care, (5) treatment initiation, and (6) viral load suppression.

A long-standing approach to reaching MSM and TW has been the use of mobile and other forms of street or venue-based outreach to engage these populations in HIV testing. These methods assume at-risk members of the population are present in sites accessible to outreach workers and often result in frequent retesting of the same individuals. Respondent-driven sampling (RDS), however, has become popular in the last decade as an alternative sampling method that can effectively penetrate deep into social networks of key populations and recruit a more representative sample [6,7]. RDS is a peer-based recruitment method that has been developed as a nonprobability sampling approach for surveys of hidden and stigmatized populations [8]. It has shown benefit in reaching individuals who may not be present at venues or who frequent health facilities less often [9]. As such, RDS may have a role beyond research in engaging MSM and TW in HIV testing and other HIV prevention and care interventions, particularly in settings of stigmatization and criminalization [10-12].

Self- or home-based HIV testing (HIVST) has also emerged as an important tool to promote HIV screening and, potentially, to increase frequency of HIV testing among key populations for whom more frequent testing is recommended [13]. Salivary-based HIV self-test kits have been approved by the US Food and Drug Administration (FDA) and are commercially available in the United States [14]. They have also been increasingly used in research and programs in developing countries as an alternative to facility-based testing [15]. One of the most beneficial aspects of HIVST is its empowerment of individuals to take control of their own health and HIV prevention or care while allowing users to test at their own convenience and in the privacy of their own home or setting of their choice.

A systematic review and meta-analysis of supervised and unsupervised HIVST in both low- and high-risk populations found that self-testing was highly acceptable, preferred by persons at high risk for HIV, and was more likely than clinic-based testing to result in partner HIV testing [15]. Self-testing has the potential to overcome the barriers experienced by MSM and TW as it relates to HIV testing uptake by placing the locus of control on the individual, increasing confidentiality, and allowing members of stigmatized groups to test in settings of privacy, safety, and dignity [16]. HIV rapid tests have grown more sensitive and less complex, making self-testing a viable alternative to clinic-based testing.

Beyond HIV diagnosis, there is considerable evidence that a major loss of HIV-infected persons in the treatment continuum occurs during disease staging [17]. In most settings, seeking and receiving clinical staging and CD4 testing is required for initiation of ART and generally requires visits to additional facilities where CD4 testing is available. This is often accompanied by long waiting times for results, concerns of unintentional disclosure of sexual or gender identity and disease status to others, and additional steps after staging to link patients to ART [18-20]. These issues around the availability of CD4 testing are common causes of losses at this critical step in the continuum.

Further, there are multiple challenges to successful navigation of the health care system, including but not limited to discrimination and stigma in health care settings. Recently, peer-navigation has been increasingly used among MSM and TW and other key populations to support navigation through a complex system of care, retention in care, and treatment adherence [21-23]. Peer navigators further provide health and HIV information and social support when coping with diagnosis, dual stigmatization of HIV and sexual or gender identity, and disclosure [21-23].

All of these challenges in the HIV care continuum are relevant in Myanmar (formerly known as Burma) and particularly so for MSM and TW populations in the country. Since the early 1990s, the HIV epidemic in the country has seen high rates of morbidity and mortality, very low access to ART, and limited and problematic HIV surveillance and reporting [24,25]. Further, the epidemic has disproportionately affected several key populations in Myanmar, including MSM and TW. While HIV rates appear to have declined among the general population, what limited data are available on key populations suggest that HIV remains concentrated in these groups, disease burdens are high, and access to voluntary counseling and testing services, prevention, and HIV treatment and care remain elusive to many [26,27]. The most recent Joint United Nations Program on HIV/AIDS and Ministry of Health report estimates that some 11.6% of the estimated 230,000 MSM and TW in Myanmar are living with HIV, with prevalence as high as 26.6% among MSM and TW in Yangon, compared to 0.53% of reproductive aged adults [28,29]. With approximately 53% estimated to be reached by prevention services and 20% reached with HIV testing, the same report highlights the need for programs that can reach MSM and TW who are not openly gay and do not seek services [28].

Barriers to HIV care have been common and chronic issues in Myanmar [26]. CD4 criterion for ART initiation in Myanmar is 350 cells/µL, and until 2014, when ART availability was limited, priority was given to patients with CD4 count below 150 cells/µL [30]. The median actual starting level of CD4 in 2012 in Myanmar was 60 cells/µL [26]. National guidelines also recommend treatment initiation at CD4 <500 cells/µL for coinfected patients and for patients from key affected populations, such as MSM and TW [31]. Recent increases in treatment access as a result of support from United States President's Emergency Plan for AIDS Relief (PEPFAR), the Three Millennium Development Goals Fund (3 MDG, a joint donor fund established in Myanmar before the Global Fund began activities in the country), the Global Fund, and the World Bank have led the National AIDS Program to estimate the availability of at least 50,000 new treatment slots in Myanmar in the next 2 years [28]. MSM and TW in Myanmar, and in many settings, are more likely to have undiagnosed HIV infection, since the stigma and discrimination they face in the community, wider society, and in health care settings can be a barrier to testing, disclosure, and seeking health care overall [32].

The primary aim of the Parasol study is to use the overarching framework of the HIV care continuum [17] to measure and overcome barriers to HIV testing and access to care through a series of 4 primary innovations for MSM and TW in Myanmar. We propose to test the following innovations to address losses in the HIV care continuum: use of RDS to reach and engage MSM and TW in HIV testing, HIV self-testing to increase testing uptake, staging of HIV disease for those who are infected through CD4 point-of-care (POC) technology [33,34], and training and capacity building of a cadre of peer health navigators for MSM and TW to increase successful health system navigation for HIV-infected MSM and TW in need of ART or HIV engagement in care [22,23,34]. Longer-term engagement in care will be assessed through the current gold standard for HIV treatment: successful HIV viral suppression as measured by quantitative viral load at 12 months posttreatment initiation [35].

The research protocol described here is a collaboration between the Center for Public Health and Human Rights at the Johns Hopkins University School of Public Health, the International HIV/AIDS Alliance Myanmar, the Myanmar Department of Medical Research, and 3 community-based organizations (CBOs): Lotus Project, Phoenix Association, and *Aye Nyien Myitta*. Funding has been provided by amfAR, the Foundation for AIDS Research.

## Methods

### Study Design

**Figure 2:**
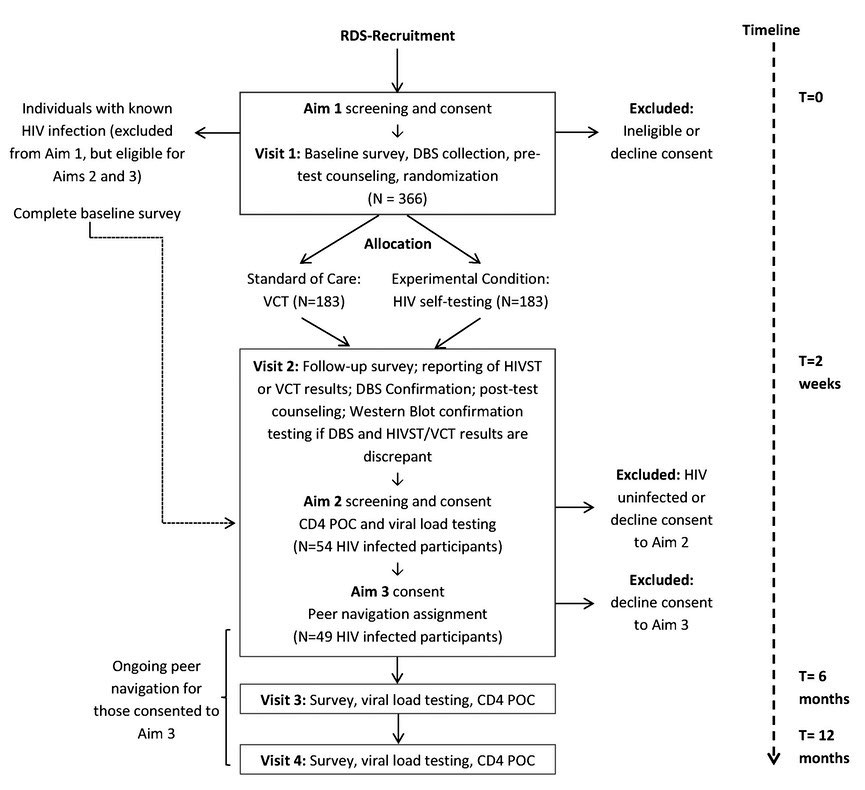
Participant flow diagram.

This study is conducted in a staged process, beginning with a qualitative, formative phase that is intended to inform further development of recruitment and intervention methodologies, followed by a 3-step continuum interventions study in Yangon, Myanmar. Specific aims and hypotheses are dedicated to each step along the continuum from engagement in HIV testing through RDS and evaluation of HIVST (Aim 1), evaluation of CD4 POC staging (Aim 2), and assessment of peer navigation for treatment initiation and adherence support (Aim 3). This manuscript describes the methodology for the continuum interventions study (qualitative methods have been described elsewhere) [36]. The participant flow diagram displays the study flow, randomization point, and target sample size (Figure 2).

### Study Site

The study is conducted in Yangon, Myanmar. Yangon, formerly the capital of Myanmar, remains an important urban center and is the largest city in Myanmar with a population exceeding 7.3 million persons in 2014 [37]. There is growing recognition and acceptance of the lesbian, gay, bisexual, and transgender (LGBT) populations in Yangon and a substantial emergence of community-based HIV prevention and care services for MSM and TW within the city.

### Aim 1: HIV Self-Testing Versus Clinic-Based Testing

To assess the effect of HIVST, we have designed a randomized trial in which MSM and TW adults in the greater Yangon metropolitan area will be recruited through RDS and offered HIV testing. Participants are randomized to receive standard clinic-based voluntary counseling and testing (VCT) or the HIVST intervention.

#### Aim 1 Study Sample

Aim 1 participants are included on the basis of the following inclusion criteria: assigned male sex at birth, aged 18 years or older, reports having had any type of sex with another man in the past 12 months, presents to the study with a valid RDS coupon (except seeds), speaks Myanmar, currently a resident of the greater Yangon area, is mentally sound and capable of providing consent to participate, and has provided informed consent to participate in the study. Participants who have been tested for HIV within the last 6 months or who are known to be living with HIV infection are excluded from participation in Aim 1. However, individuals who disclose a positive HIV status when Aim 1 eligibility is determined are considered for enrollment in Aims 2 and 3, provided they meet all eligibility criteria for those aims. While there are emerging and increasingly open gay communities in Yangon, the traditional categories of sexual orientation and gender identity in Myanmar culture are blurred; locally, the term MSM is often used interchangeably across male and transgender-identified populations and these groups often attend similar community-based events [38]. Thus, this study focused on the inclusion of both MSM and TW populations.

The target sample size needed for the RDS sample is 366 MSM and TW participants. This estimate achieves 2 goals. First, it allows for a comparison of HIVST versus clinic-based HIV testing. Assuming an error of .05 and 80% power to detect a 15% difference in acceptability between the 2 testing methods (60% acceptability of clinic-based testing and 75% acceptability of self-testing), a sample of 330 (or 165 per group) is needed. Upon factoring a 10% nonresponse in both testing methods, an effective sample size of 366 MSM and TW participants is calculated. Second, this allows for subsequent enrollment of MSM and TW participants into Aims 2 and 3. Assuming a conservative 15% prevalence of HIV among the sample [28], this identifies 54 participants who are then eligible for Aims 2 and 3.

#### Aim 1 Recruitment

Participants are recruited via RDS, given demonstrated benefits to (1) potentially recruit a more representative sample and (2) reach deep into social networks to recruit participants who are often not reached through outreach, venue-based, or snowball sampling methods for engagement in HIV testing [6,7,9]. In this method, 8 to 10 MSM and TW seeds will be recruited by study investigators. Seeds, who begin recruitment, are individuals who represent a range of characteristics and are well-networked within the MSM and TW population. The study team works with local CBO partners in Yangon to identify MSM and TW seeds. Seeds complete the first visit study activities and are then given a maximum of 3 coupons with which to recruit their peers from within their social or sexual networks. These peers are invited by the seed to present to the study office with their coupon for participation in the study. If eligible and after completing baseline study activities, the recruited participant may, in turn, become a recruiter. Participants who agree to become a recruiter undergo recruitment training and receive a maximum of 3 coupons for distribution. This process continues until the sample size has been reached. This sampling method has been successfully used by the research team for several research studies of HIV among MSM, including in Malawi and Russia [7,39,40]. RDS has also been used by other investigators to research HIV among people who inject drugs, MSM, and TW in Myanmar [41,42].

**Figure 3:**
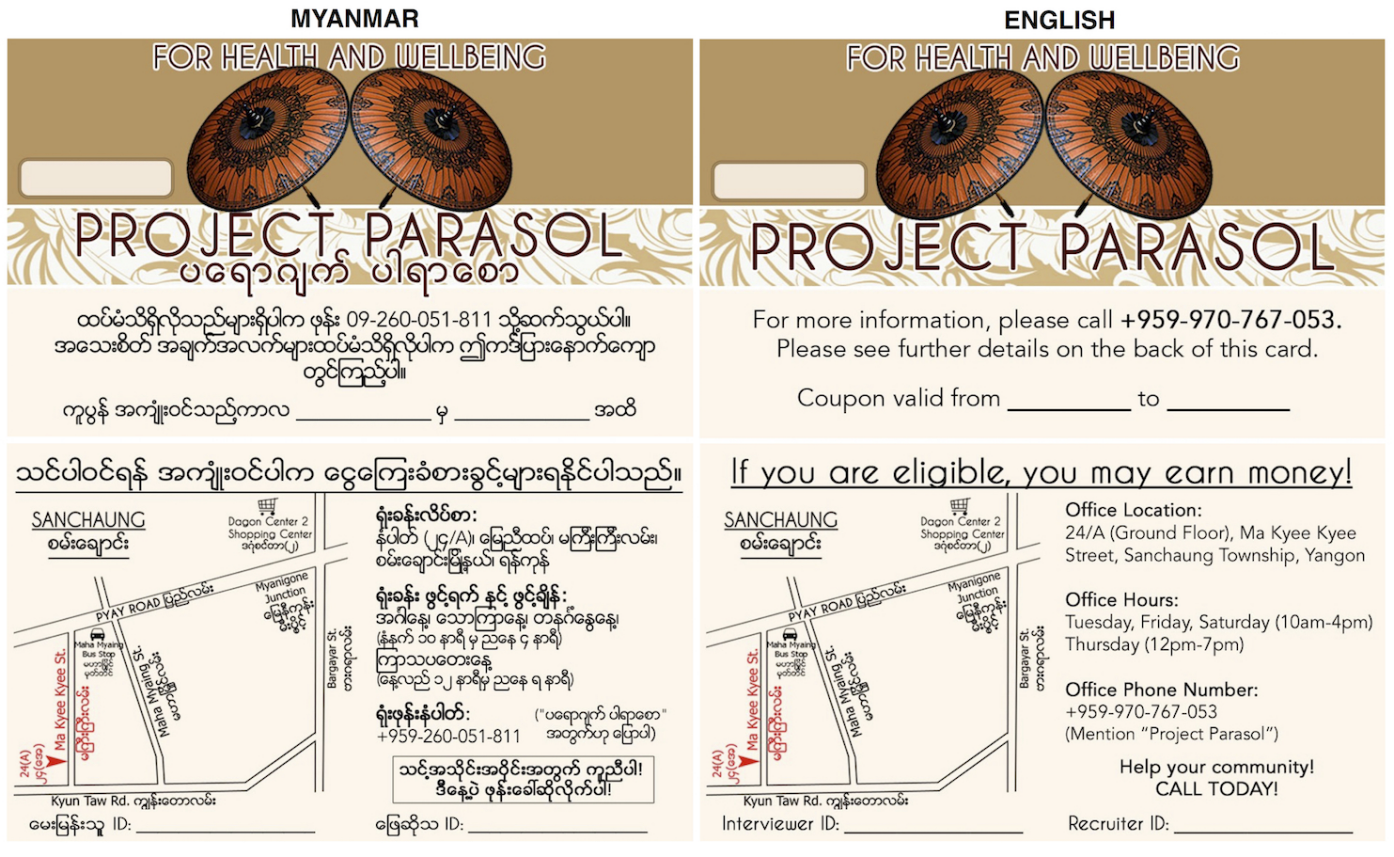
Respondent-driven sampling coupons (front and back).

Recruitment coupons are prenumbered with a unique coupon number prior to distribution (Figure 3). Coupons contain other information including the study telephone number, site operating hours, and coupon expiration date. There is no information on the coupon that could be used to identify the holder as MSM or TW and no personal information is recorded on the coupon. Coupons are collected from recruited candidate participants at the study site at the time of eligibility screening and the returned coupon number is recorded. Coupons allow for the research team to trace the recruitment process and create analytical weights for the sampling process.

#### Aim 1 Data Collection

Eligible participants recruited through the RDS are verbally consented using paper-based consent forms in private rooms within the study office. Upon providing consent, participants complete a structured, 30- to 45-minute interviewer-administered, tablet-based questionnaire, which serves as the baseline assessment. The questionnaire allows for comparison of HIVST versus clinic-based testers and attrition analysis of those who return versus do not return for their follow-up visit. Measures in this questionnaire include demographic and network characteristics, previous testing history and experiences, sexual and substance use risk profiles, access to HIV and sexually transmitted infection prevention and care, measures of perceived and experienced stigma in health care settings, concerns over police or other security harassment, and factors related to cost, distance, convenience of testing venues, and acceptability of assigned testing method. Individual network size measures, commonly collected for the analysis of RDS data, assess the number of MSM and TW in the Yangon area the participant knows and has seen or spoken to in the last 6 months. The questionnaire also includes 2 of 5 parameters recommended by Pant Pai et al [15] for HIVST assessments, specifically acceptability of testing methods and motivations for HIVST among users.

Following the questionnaire, pretest counseling is administered prior to collection of dried capillary blood spot collection (DBS) via a finger prick. DBS is analyzed at a local reference lab in Yangon. The primary purpose of DBS collection is to ensure that the study team receives HIV test results for any participants who do not return to the clinic for their follow-up visit. The DBS will also confirm clinic-based HIV test or preliminary HIVST results during visit 2, allowing for further validation of the self-testing and clinic-based results.

Following pretest counseling, participants will be electronically randomized to clinic-based VCT or HIVST. Participants assigned to VCT are provided with referral information for partner clinics and instructed to complete clinic-based HIV testing services within 2 weeks. Individuals randomized to HIVST will be provided with the OraQuick in-Home HIV Test (OraSure Technologies) self-testing kit and instructions for use. OraQuick in-Home HIV Test is an FDA-approved oral HIV test that allows the user to self-administer the salivary test in private and displays results within 20 minutes. The OraQuick in-Home HIV Test tests for HIV-1/2 antibody test and has a sensitivity of 91.7% and specificity of 99.9%. The test is recommended for use as a method to screen for HIV infection to be followed with confirmatory testing. Johns Hopkins University (JHU) has partnered with OraSure to provide Myanmar language instructions within the kit. Participants randomized to HIVST are instructed to complete HIVST any time before their scheduled second visit. HIVST participants are provided with a study telephone number that they can call for assistance at any time of day.

All participants are instructed to call the project office immediately if they receive a preliminary positive HIV test result. Study staff schedule these participants to return to the project office for visit 2 as soon as possible. This ensures that all participants are offered posttest counseling, confirmatory testing, and enrollment in Aim 2 (CD4 POC staging) as quickly as possible. DBS analysis is expedited for participants who cannot be reached or who do not disclose their HIV testing results. HIV testing is considered complete when participants have received results from their assigned method of testing and report awareness of their HIV status.

#### Aim 1 Follow-Up

All participants are scheduled for a second visit 2 to 3 weeks after the primary visit. During this visit (visit 2), self-reported VCT and HIVST results are ascertained. Analysis of DBS specimens is conducted between visit 1 and 2 to serve as confirmatory testing of results identified through clinic-based VCT or HIVST. Special effort are made to recontact, counsel, and provide appropriate referrals for participants who do not return for the follow-up visit but who are identified with HIV infection during analysis of DBS. Participants found to be preliminarily HIV-negative are given posttest counseling, which includes an explanation of the test result, advice on risk reduction, and the provision of condoms, lubricant, and referrals. These participants are advised to seek HIV testing again in 3 months and are provided with contact information for local nongovernmental organizations who provide HIV testing and care services to MSM and TW. Participants confirmed HIV infected are given posttest counseling and offered enrollment in Aim 2 to conduct immediate CD4 POC staging. Participants who decline enrollment in Aim 2 are provided with referrals for standard CD4 testing as well as treatment and care services. Participants with discordant DBS and self-reported HIVST or VCT results are asked to provide an additional blood sample for confirmation by Western blot.

All participants are asked to complete a structured, 30-minute interviewer-administered questionnaire during visit 2 that serves to evaluate uptake and acceptability of clinic-based or HIVST methods. Participants also receive secondary reimbursement for recruitment of peers at the second visit, which is a standard practice of RDS and will incentivize this follow-up visit.

#### Aim 1 Analytical Approach

Baseline characteristics are analyzed using a program written for Stata (StataCorp LLC), which makes statistical adjustments for RDS sampling to characterize unbiased asymptotic estimates of disease burden and other characteristics by weighting for social network size (RDS-II estimator) [43]. Exploratory analysis is conducted to evaluate recruitment depths and potential biases induced by recruitment [7]. Results include descriptive statistics related to sociodemographic characteristics, knowledge of STI/HIV, and sexual behavior and attitudes of MSM and TW populations. Bivariate and multivariable analyses using multiple logistic regression or log binomial regression, depending on the calculated prevalence [44], are performed to determine the association between HIV infection and key behavioral outcomes and other variables. Additional analysis focuses on the parameters recommended by Pant Pai et al [15] for HIVST assessments, conducting a stratified analysis of acceptability of testing methods, accuracy, concordance with health care worker testing, feasibility and motivations for testing among consumers, and comparability of HIVST to VCT.

The primary hypothesis for this continuum innovation step is that a higher proportion of persons who are randomized to HIVST will complete HIV testing than those randomized to standard clinic-based testing. Given the randomized nature of the study design, the study team will use a standard comparison approach to assess the outcomes of the 2 intervention arms for this aim. Standard contingency tables with Fisher exact test for significance is used to evaluate the efficacy of the intervention. A comparison of intervention arms (HIVST vs clinic-based) is the primary outcome measure, and balance in potential measured confounders is assessed. Implementation measures of HIV testing include uptake of HIV testing services, fidelity of the implementation of the intervention, and acceptability of HIV testing options.

Attrition analysis is conducted to assess the potential differential loss to follow-up by individual characteristics (demographics, sexual identity, and distance from office/clinic) as well as assigned intervention groups. Where possible, we attempt to recontact participants to understand individual reasons for failed return, which is particularly important during the start-up of the interventions, in which logistical changes can be made to address losses to follow-up. Any logistical changes are documented.

### Aims 2 and 3: CD4 Point-of-Care and Peer Navigation

#### Aims 2 and 3 Design

Participants diagnosed with prevalent or incident HIV infection during participation in Aim 1 of the study are offered CD4 POC to assess whether this technology can increase the likelihood that HIV-infected MSM and TW are successfully staged for HIV disease and engaged in care (Aim 2). All persons who accept CD4 POC testing are offered peer navigation and viral load testing in the final stage (Aim 3). Treatment adherence and clinical outcomes, including the proportion of MSM and TW participants who achieve successful viral suppression, is assessed by nonrandomized comparison of participants who accept and decline peer navigation support. Participants of Aims 2 and 3 follow the same procedures; thus, the following section jointly describes the methodology for Aims 2 and 3.

#### Aims 2 and 3 Recruitment

Participants who test positive during Aim 1 or who report a previous diagnosis of HIV infection are offered enrollment by the study team into Aims 2 and 3. Separate consent forms are provided for each of these aims. Specifically, the consent form for Aim 2 requests consent to participate in CD4 POC testing, and the consent form for Aim 3 will request consent for participation in peer navigation.

#### Aims 2 and 3 Study Sample

Inclusion criteria for Aims 2 and 3 are assigned male gender at birth, aged 18 years or older, reports having had any type of sex with another man in the past 12 months, is mentally sound and capable of providing consent to participate, speaks Myanmar, current resident of the greater Yangon area, diagnosed with HIV infection, not currently engaged in any treatment or care programs, and has provided informed consent to participate in the study. Participants will be excluded from Aims 2 and 3 if they report that they are already linked into an HIV treatment or care program or have tested negative for HIV infection.

The overall study sample size estimate was driven by the numbers needed for the feasibility and acceptability studies of CD4 POC testing, viral load, and peer navigation, with a target of 54 participants in CD4 POC testing to assess key parameters. Assuming a 10% decline in participation for peer navigation and viral load testing, our target sample for viral load and peer navigation is 49 participants. As data analysis and monitoring are ongoing during data collection, RDS can be extended for additional recruitment should additional recruitment be required to reach targets in Aims 2 or 3. Further, if necessary to reach our desired Aim 2 sample size, additional MSM and TW who are newly HIV-diagnosed can be recruited from partnering organizations for this phase.

#### Aim 2 CD4 Point-of-Care

CD4 POC testing capacity is available at the study site. Given the need for CD4 POC testing, there is no randomization of participants during this phase of research. We use the BD FACSPresto Near Patient CD4 Counter (BD Biosciences) system for POC CD4, which is currently available but in limited use in Myanmar. This system uses the BD FACSPresto cartridge, which contains desiccated reagents that eliminate requirements for a cold chain and cold storage and is well-suited to rural areas and settings with unstable power. The BD FACSPresto Near Patient CD4 Counter system was selected given its availability in Myanmar and limited use by local nongovernmental organizations in Myanmar to provide free CD4 POC services to key populations who have been diagnosed with HIV infection. The system is portable and can provide easily read CD4 outputs within 20 minutes, while patients wait to receive their results. The BD FACSPresto machine contains a built-in training module along with a detailed manual for easy training and operation. In addition to this, laboratory staff attended a training provided by Yee Shin Co Ltd, the Yangon-based regional distributer of the machine and cartridges. Yee Shin Co Ltd is also available to the staff for troubleshooting and other maintenance services as needed. Prequalified by WHO, the BD FACSPresto Near Patient CD4 Counter system has demonstrated reliability and accuracy when used in resource-limited settings [45-47]. This innovation can reduce continuum losses through immediate provision of results, post-CD4 stage counseling, and access to treatment for those who meet treatment criteria on the day of testing. Persons with very low CD4s can thereby be immediately engaged with peer navigators (Aim 3) to seek ART access.

#### Aim 3 Peer Navigation

If successful, increasing HIV status awareness through HIVST and POC CD4 will identify a significant number of persons in need of health care and ART. All Aim 2 participants are offered enrollment in Aim 3, which intends to address barriers to HIV care and ART adherence by comparing those who opt into peer navigation to those who decline.

Peer navigation is offered to all participants on the account of high rates of existing AIDS-related morbidity and mortality among MSM and TW populations living with HIV in Myanmar. For this phase of the project and given the severity of clinical needs, we will not conduct a randomized assessment of peer navigation. Consequently, we measure actual numbers of persons who successfully start on ART, comparing those who opt into peer navigation to those who decline. We treat this aim as an observational study, with peer navigation assistance as the exposure of interest and ART uptake as the outcome. All eligible participants are offered peer navigation with approximately 8 participants assigned to each peer navigator.

Trained peer navigators follow and support all clinically staged participants who have accepted navigation support for 12 months. Peer navigators offer focused training on the importance of CD4 and viral load monitoring, self-care when living with HIV, and methods to prevent HIV transmission. Participants are given contact information for their assigned peer navigator and are encouraged to reach out to them for assistance navigating the Myanmar health system. The role of the peer navigator is to provide support and information to HIV-positive MSM and TW who are seeking HIV treatment and care in the Myanmar. In order to maintain confidentiality in this peer relationship, the questionnaire administration and appointment reminders are the responsibility of office staff.

Peer navigators are selected from the MSM and TW population by Alliance and partner CBOs. Ideally, peer navigators will have prior experience working in HIV prevention or other outreach, but this is not required. Peer navigators undergo an intensive training on topics of MSM and TW health, HIV prevention and care, disclosure of HIV status and issues of social support, and other related topics. Training will build on past training used locally as well as in Malawi and South Africa and incorporate key aspects from the Fenway Guide and other validated MSM training materials [48,49]. Local CBOs and MSM and TW participants also provide input to trainings to ensure contextual relevance. Peer navigators are provided with condoms and lubricants for distribution as well as informational materials on where specific (nonstigmatizing) services can be obtained. Peer navigators are overseen by the research coordinator and report on a weekly basis for debriefing. The research coordinator occasionally monitors participant meetings to ensure fidelity to the curriculum and adherence to the study protocol.

#### Aims 2 and 3 Data Collection

**Figure 4:**
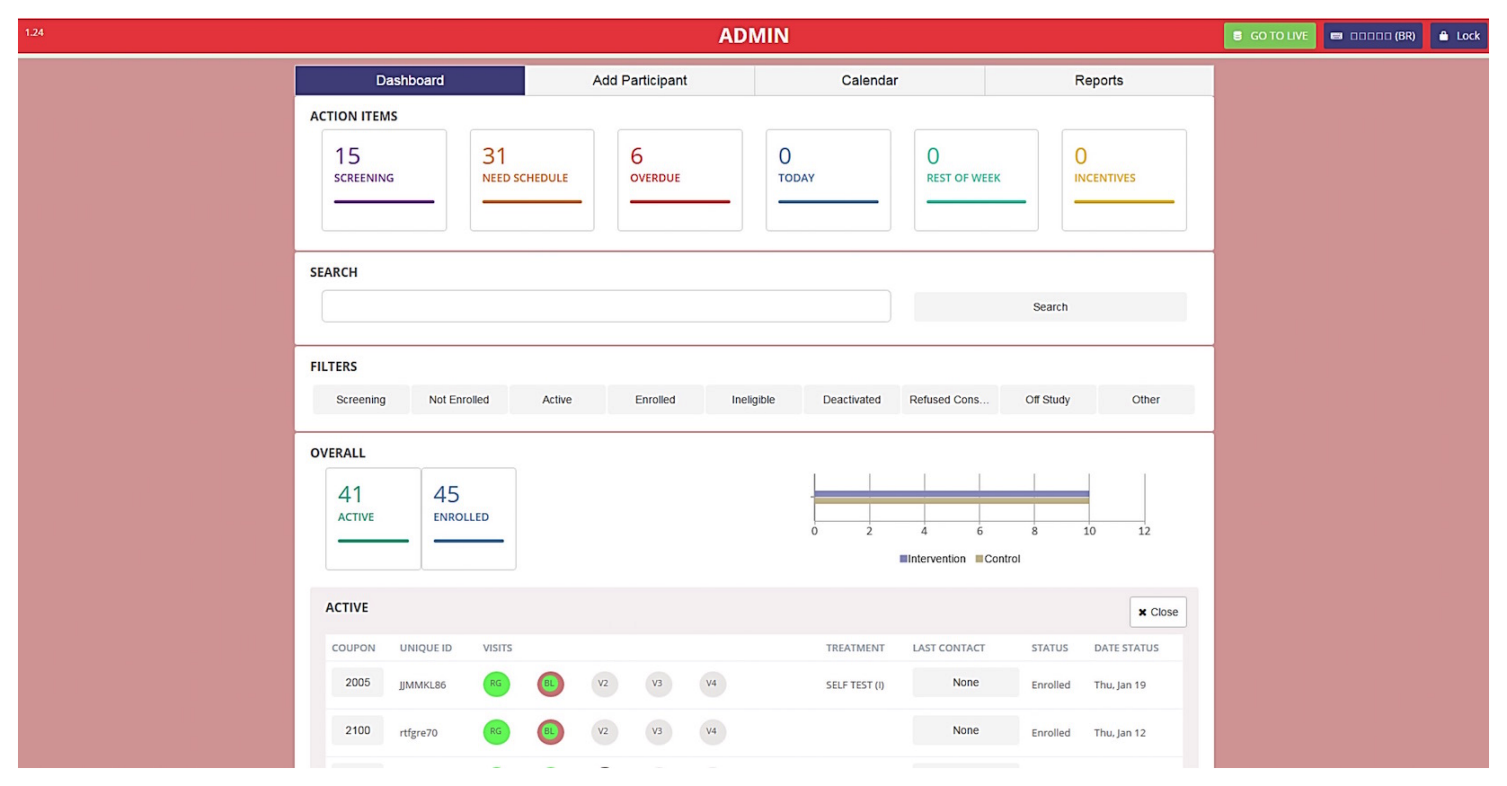
Participant tracking system (English).

**Figure 5:**
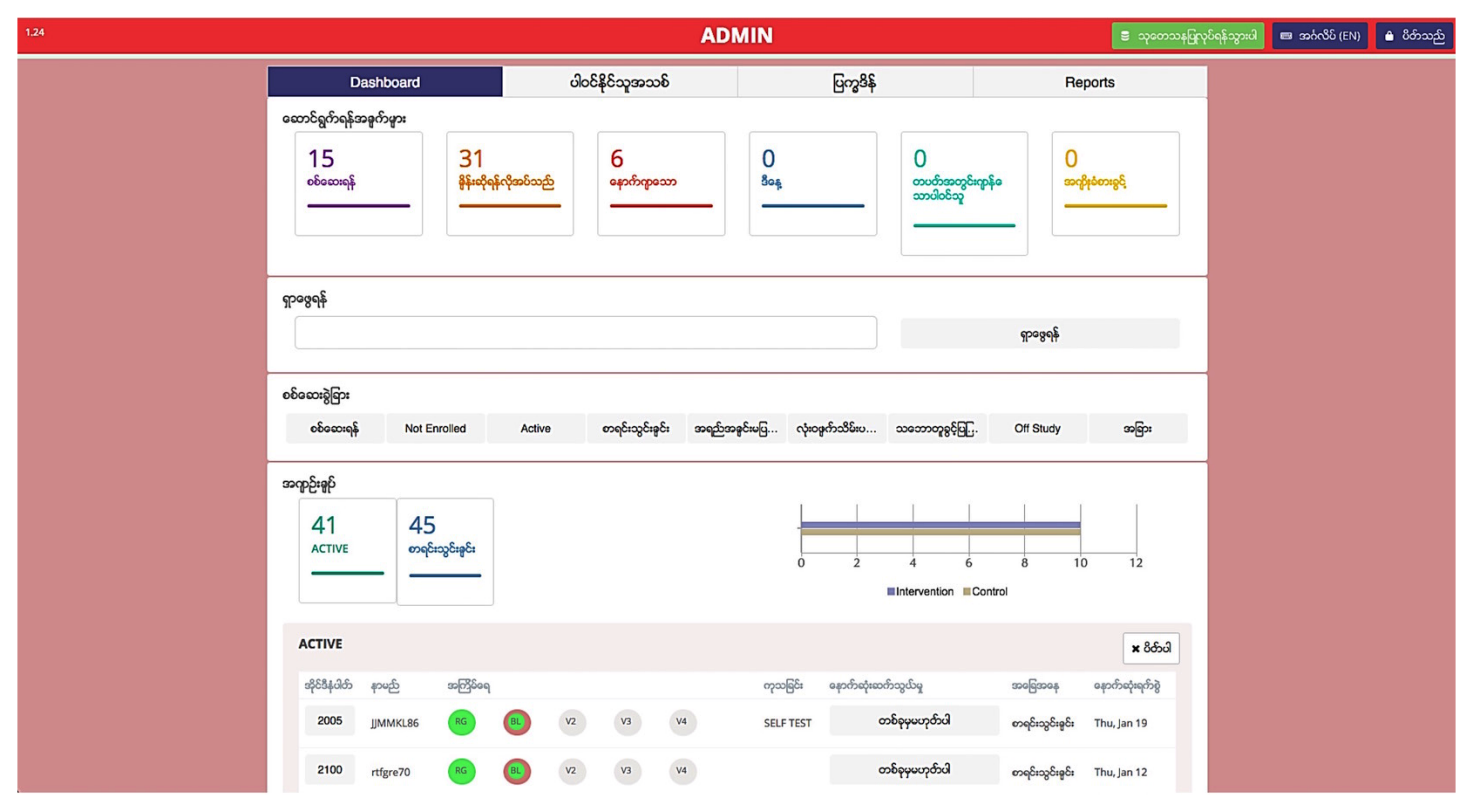
Participant tracking system (Burmese).

Upon determination of eligibility and verbal confirmation of informed consent, participants complete a structured 20-minute interviewer-administered questionnaire. Participants are then asked to return to participate in surveys conducted at 6 and 12 months. Surveys assess the primary outcome and initiation of ART as well as ART adherence (among those successfully initiated) and other clinical outcomes, including viral suppression, as measured by HIV viral load assessment at 0, 6, and 12 months. This questionnaire is used to assess the strength of peer navigation in improving access and uptake of viral load by MSM and TW living with HIV in care. Peer navigation implementation data (eg, number of meetings per participant, types of support provided) along with participant perceptions will be collected to understand the benefits and challenges of peer navigation and note opportunities for improvement. Incentives are distributed at the conclusion of each follow-up survey. An electronic data entry and tracking system has been developed for this study and allows for registration, screening, survey data collection, data entry of laboratory results, and participant tracking through a secure, Health Insurance Portability and Accountability Act–compliant site. This system is used throughout all phases of the study (Figures 4 and 5).

#### Aims 2 and 3 Analytical Approach

The primary hypothesis for Aim 2 is that a higher proportion of MSM and TW living with HIV who accept CD4 POC testing will be successfully staged. The primary hypothesis for Aim 3 is that HIV-positive persons who accept peer navigators will have a higher likelihood of achieving ART treatment access than those who decline peer navigation. Aims 2 and 3 are analyzed as observational studies, with CD4 POC testing and peer navigation assistance as the exposures of interest and ART uptake and viral suppression as the outcomes. Specifically, the primary outcomes of interest follow the HIV care continuum and include the actual numbers of persons who accept CD4 staging, are linked to treatment, successfully initiate ART, accept viral load testing and peer navigation, reach viral suppression, and are lost to follow-up.

We conduct longitudinal analyses to measure the associations between these innovations and impact outcomes, including time to CD4 staging, receipt of CD4 results, time to engagement in HIV care and treatment for those eligible, barriers and facilitators to completing CD4 staging, perceived and experienced stigma in health care settings, and uptake of treatment referrals. Regression analysis controls for potential confounding by participant demographics and behaviors. Additional analyses related to peer navigation assess frequency of peer navigator meetings, types of discussions and HIV prevention materials provided, and associated changes in ART adherence and viral load testing. Statistical analysis to achieve Aims 2 and 3 will be conducted using multilevel logistic regression to allow for exposure to peer navigation to vary by time. This will be implemented using regression models specified for proportions in Stata 14 (StataCorp LLC). Longitudinal analysis compares time to CD4 POC and ART (among those eligible) for participants testing positive for HIV infection, controlling for other potential confounders.

#### Human Subjects Considerations

Discussion of HIV and sexuality is inherently sensitive in nature. As such, all study activities are conducted with emphasis on confidentiality and privacy of participants. The study office provides the participants with a safe environment free from outward harassment or discrimination. Our collaboration with the International HIV/AIDS Alliance Myanmar and local CBOs serving the MSM and TW populations ensures that all activities are sensitive to stigma and other challenges faced by MSM and TW and designed interventions are acceptable to the population. The surveys and data collection are conducted in a private room located in the study offices. Study staff members are required to complete human subjects training and provide their certificates of training prior to conducting human subjects research. All study activities are conducted in private with individual participants; no individual other than the interviewer or laboratory technician is permitted in the room at the time of data collection. During data collection, the participant has the right to refuse to answer any questions he or she is not comfortable answering to protect privacy.

Trained Alliance staff working on this project obtain informed consent from participants but allow participants to leave a mark in lieu of their signed name. All consent processes use a Myanmar language consent script, approved by Johns Hopkins School of Public Health Institutional Review Board and the local ethical review board. During the consent process, the staff member explains the study in detail, outlining the purpose, sequence of events, rights, potential risks and benefits to participants, and eligibility criteria. This information, along with a local study phone number that can be called with questions or concerns, is available on a paper version of the approved version of consent script. Study staff read aloud the consent form and will allow participants to ask any questions at any time prior to requesting consent for participation in the study. The consent process, along with surveys and biologic specimen collection, will be conducted in private offices to maintain participant confidentiality and privacy.

Study staff keep confidential phone numbers and first names of potential participants for scheduling purposes only. One hard copy of the contact list is stored in a locked cabinet and an electronic version is saved in the electronic tracking system, separate from study data. Only staff members tasked with scheduling participants have access to the electronic list, by permission of the in-country principal investigator. The staff are instructed not to leave any voice or text messages with participants, to verify the identity of the individual, and to not mention any potentially stigmatizing behavior or HIV status during the phone conversations. When the study is mentioned over the phone or while reminding participants of an appointment, it is referred to as a health and wellness project.

Unique identifiers will be used to link study data and are constructed for all participants using information known only to the participant; these will be created to link all surveys, specimens, and laboratory results. The code is formulated from questions that are easily answered, reproducible, culturally appropriate, individually unique, and that MSM and TW will be comfortable answering. The unique identifier is an 8-digit reproducible alphanumerical code developed using the using the first 2 letters of the participant’s last name, the first 2 letters of the participant’s first name, the first 2 letters of the participant’s father’s first name, and last 2 digits of the participant’s birth year. Burmese names are Romanized to English letters based on a transliterated chart of the Myanmar alphabet for purposes of creating a unique ID. During the process of creating the unique ID code with the participant, the study team member indicates that the participant only needs to respond with the appropriate numbers or letters to the question and do not need to give the full response (eg, participant only needs to provide the first letter of the city where he or she currently lives rather than state the full name of the city). Doing so provides an additional layer of privacy for the participants. This unique identifier can be recreated throughout the study and will facilitate linking study participants with biological results while maintaining anonymity. This process has been successfully used in past research studies conducted by this study team [39,50].

All surveys are interviewer-administered and collected using secure, computer-based systems. Anonymous, paper-based surveys are used only as back-up to the computer-based systems. Any anonymous, paper-based data collection forms are secured in a locked cabinet in the project offices. Data entry will take place at the study office, where study data are maintained on designated password-protected computers. Electronic transfer from the study office to JHU occurs only through encrypted files. The JHU principal investigator (CB) and in-country principal investigator (SN) control access to the data. Only relevant study staff who have completed ethical training and have permission from the principal investigator have access to the data.

Following completion of study activities, eligible participants receive 9000 kyat (about US $7) at visit 1 as incentive and reimbursement of travel costs to the project office. Participants who are ineligible at visit 1 also receive 1000 kyat (about US $0.80) for travel expenses. Participants are provided with another 5000 kyat (US $4) at visit 2 for travel expenses as well as an additional 1000 kyat (about US $0.80) for each RDS recruitee who is eligible and participates in the study (maximum of 3 recruitees). These incentive amounts are consistent with other RDS studies implemented in Myanmar and are determined to encourage recruitment without unduly coercing participation [51]. For eligible participants, subsequent visits at 6 and 12 months upon enrollment into Aims 2 and 3 include 10,000 kyat (about US $8) as incentive and to cover travel expenses.

## Results

Formative, qualitative research was completed between June and September 2015. This included 12 MSM and 13 TW in-depth interview participants, as well as 12 MSM and TW participants and 23 service providers and community leaders participating in focus group discussions [36].

Participants provided logistical input, particularly as it related to location of the study office, recommending that the office be in a neighborhood in the center of the city and directly accessible by most bus lines. Participants also provided insights into social considerations for providing HIV testing, prevention, and care for MSM and TW populations in Yangon; opinions, preferences, and concerns about HIVST; and experiences and perceptions of HIV treatment and care. Generally, both MSM and TW described concerns about discrimination and stigmatization of MSM and TW populations within government health systems, often preferring to use nongovernmental community-based services that are an expanding presence in Yangon. Regardless of general perceptions that MSM and TW were at high risk for HIV acquisition and emerging options for HIV testing and care, MSM and TW participants described hesitation about HIV testing that was often related to fear of being seen in a testing facility that would unintentionally disclose one’s HIV status, gender, or sexual identity.

The privacy and convenience offered by HIVST garnered significant interest from a majority of focus group participants, many of whom indicated the appeal of using these kits in the privacy of their own homes. This interest was weighed, however, against concerns that incorrect use could produce an inaccurate result and that lack of pretest counseling could lead to unintended adverse effects such as suicide by someone who receives a positive result. A further concern was that users would not be effectively linked to care following a positive diagnosis. These findings led to concrete protocol changes for the subsequent self-testing intervention. Specifically, in collaboration with OraSure, we developed new Myanmar language instructions for use, included a sticker on all packaging that provided a 24-hour telephone number in the case of questions or concerns, and included provision of pretest counseling for participants that included linkage to services when participants were provided with the self-test at the study office.

MSM and TW qualitative participants also described a variety of challenges in moving through the HIV care continuum. Despite a significant increase in availability of ART in Myanmar in the past 5 years, the majority of participants reported issues with staging, treatment, and care, which can delay treatment initiation and inhibit adherence. Participants expressed frustration with current health system structure; many described having to visit different clinics and hospitals for testing, staging, treatment, and monitoring as well as having repeated confirmatory tests at each provider they visited.

MSM and TW participants expressed a strong preference for treatment and care services that are friendly, discrete, and convenient. Long travel times and cost, limited clinic hours, and unfriendly staff were described as barriers by many participants, especially when they are required to visit different providers for testing, staging, and treatment. In response, the research protocol was adjusted to include night and weekend office hours, the project office was chosen because of its central location and proximity to bus lines, and MSM and TW office staff were hired to create a nonstigmatizing and welcoming environment.

Qualitative participants also voiced concerns that home visits by peer navigators could expose their HIV status to their family or the community. To protect the privacy of the participants who enroll in peer navigation, we adapted the protocol to allow peer navigators to visit participants in any mutually agreeable location. This flexibility allows the participant to determine if, how, and when they would like to disclose their status to family and friends and minimizes the chance of unintentional disclosure.

Following the qualitative research, Aim 1 recruitment began in November 2015 with subsequent enrollment into Aims 2 and 3, and study activities for the 3 aims are currently ongoing. Broader social changes have since led to further modification of the above protocol. First, during the development of the intervention protocol the government of Myanmar adjusted the ART guidelines to reflect the importance of early detection and treatment in key populations at risk of HIV infection [52]. Recent updates to the WHO guidelines have since prompted the government to change this strategy and promote immediate ART initiation for all key populations, including MSM and TGW, regardless of CD4 count [28,53]. Our protocol had initially focused on the inclusion of ART-eligible MSM and TW in Aims 2 and 3; however, these policy changes made all newly diagnosed participants eligible for ART. Thus, the research plan was adjusted to incorporate this change, and all participants found to be living with HIV are referred to treatment and care programs and offered peer navigation assistance.

Throughout the implementation of this study, many nongovernmental organizations in Yangon have begun to offer home visits and peer support programs for clients who are on ART. Given the recent popularity of such programs and potential competition between this study and ongoing programs, we further altered the procedures in Aim 3, allowing participants to use our peer navigation services or, alternatively, use external peer navigation services from organizations providing the participant’s ART. Additional survey items were subsequently developed to capture data about types and frequency of services participants receive from these organizations to use in the comparison of peer navigation versus declined navigation.

## Discussion

This study seeks to identify successful and acceptable methods to address losses in the HIV care continuum among MSM and TW in Myanmar. Formative, qualitative research has been completed and was used to further refine the study protocol and interventions. Qualitative research identified several perceived benefits and concerns related to HIVST, which allowed us to ensure instructions of HIVST were understandable to participants and that participants had appropriate pretest counseling prior to HIVST and were linked to counseling and services following testing. Additional identification of barriers and facilitators to HIV care, ART initiation, and adherence during qualitative research allowed for further refinement of the intervention and study procedures. Following the completion of the formative research, enrollment into Aim 1 began in November 2015 with subsequent enrollment into Aims 2 and 3 with prospective follow-up for participants with biologically confirmed prevalent or incident HIV infection and who had not previously been engaged in care. These participants are being followed to determine the impact of CD4 POC and peer navigation on ART initiation and virologic suppression.

Early identification of HIV infection, engagement in care, and ART adherence are critical components to provide both therapeutic benefits for people living with HIV and to reduce onward transmission of HIV among at-risk populations [54]. The National AIDS Program in Myanmar has recognized the urgent need to respond to the HIV epidemic that is concentrated among MSM, TW, and other key populations; however, innovative methods to overcome stigma and provide access to HIV testing and care services are needed. The growth of the nongovernmental organization community in Myanmar, coupled with the increasing implementation of and capacity for innovative methods, can very well support these national efforts and may have aggregate effects against the HIV epidemic among MSM and TW.

## Appendix

Multimedia Appendix 1 Reviewers' comments.

## Abbreviations

3 MDG: Three Millennium Development Goals Fund
amFAR: Foundation for AIDS Research
ART: antiretroviral therapy
CBO: community-based organization
DBS: dried capillary blood spot collection
FDA: Food and Drug Administration
HIVST: HIV self-test
HPTN: HIV Prevention Trials Network
JHU: Johns Hopkins University
LGBT: lesbian, gay, bisexual, and transgender
MSM: men who have sex with men
PEPFAR: United States President's Emergency Plan for AIDS Relief
POC: point-of-care
RDS: respondent-driven sampling
TW: transgender women
VCT: voluntary counseling and testing
WHO: World Health Organization
