# Supplementary material for: The Parasol Protocol: An Implementation Science Study of HIV Continuum of Care Interventions for Gay Men and Transgender Women in Burma/Myanmar
Source: JMIR Res Protoc. 2017 May 17;6(5):e90. doi: 10.2196/resprot.7642 (PMC5451637; doi:10.2196/resprot.7642)
Supplement: Multimedia Appendix 1 [file resprot_v6i5e90_app1.pdf]

THE GMT INITIATIVE amfAR

Implementation Science Research

Phase 2 Concept Paper Review

Principal Investigator: **Chris Beyrer, MD, MPH**
PI Institution: **Johns Hopkins Bloomberg School of Public Health**
Project Title: **Continuum of Care Innovations for GMT in Burma/Myanmar**

Review Questions:

1. The proposed intervention(s), if successful, could have a substantial impact on important gaps in the prevention and/or treatment cascade(s) for GMT in low- or middle-income settings.
2. The proposed service delivery model applies proven interventions in new ways to address the HIV service needs of GMT in low- or middle-income settings.
3. The proposed study design uses sound implementation science methods to increase our understanding and application of service delivery models.
4. The proposed program is feasible to be implemented, assessed, and disseminated in the allotted, three-year time period.
5. The proposed interventions have the potential to be replicated and scaled in other low- and middle-income settings for GMT populations.
6. The proposed research team (individuals and institutions) possess the expertise, experience, and resources required to successfully implement and complete the study.
7. The proposed research team includes a balanced partnership between the research organization(s) and at least one community-based organization or clinic that is led or largely staffed by GMT and serves a GMT population in at least one low- or middle-income country.

**Question →**	**1**	**2**	**3**	**4**	**5**	**6**	**7**
	1. Agree very strongly	2. Agree	2. Agree	2. Agree	1 Agree very strongly	1. Agree very strongly	2. Agree
	1. Agree very strongly	1. Agree very strongly	1. Agree very strongly	1. Agree very strongly	1 Agree very strongly	1. Agree very strongly	2. Agree
	3. Agree with reservations	3. Agree with reservations	2. Agree	3. Agree with reservations	3. Agree with reservations	1. Agree very strongly	2. Agree

REVIEWER COMMENTS	
	This is a very strong proposal by a very experienced team. It will provide insight into 3 stages of the care cascade: 1. testing (by using home testing); 2. staging (by using POC CD4); and 3. linkage (by using peer navigators). The design seems well-thought out in implementation and in analysis. Some issues: 1. The first randomization will presumably only enroll pts willing to be HIV tested and perhaps willing to go either to clinic or do home testing. This may not get at the GMT who would only do one or the other. 2. How will HIV positives be detected from home testing? 3. I'm not clear on the logistics of the CD4 POC vs. clinic testing. If only some clinics have CD4 testing, will those without testing be included in the study? 4. If Burma only allows ARVs at CD4 of <150, will this proposal address this deficiency in any way? What is done with the GMT with CD4 150-350? What is done with the >500 group? 5. Can HIV positives out of care found on the initial RDS sampling be recruited directly into the 2nd randomization? 6. Sample size calculations aren't clear to me.
	Strengths:<br>-Highly experienced investigators<br>-Well written proposal with clear aims<br>- proposal addresses multiple steps of care continuum<br>-Use of innovative (for the region, and for other low/middle income countries) technologies such as self testing and POC CD4 testing has the potential to impact significantly on the care continuum.<br>-This study will increase knowledge about attitudes and barriers to testing for GMT in Myanmar<br>-POC tests are suited to the environment - issues of cold chain addressed by investigators<br>-If proven, these appear to be feasible interventions for roll out in other settings<br>-High level of collaboration expected with GMT CBOs, has potential to significantly improve research capacity<br><br>Questions: Not clear how staff will be trained to deliver CD4 testing & whether this testing will be done by clinical or non-clinical (CBO or patient navigator) staff, please clarify.<br><br>How/over what time period will peer navigators be identified, recruited and trained? This was not in the timeline.<br>What concerns do investigators have in terms of using peer navigators within these communities with high levels of stigma
	This project shows a strong and concrete partnership with MSM community networks in Myanmar and promising approach to recruit people into HIV testing. However, the project does not mention about the limitation of ARV in Myanmar since supply for ARV in Myanmar is still in question. If people are aware of their zero-status but do not have medications, what would be a solution to this or how this project can ensure that GMT who are in need to ARV will definitely have access to ARV.
